# Survey of sand fly fauna in six provinces of Southern Vietnam with species identification using DNA barcoding

**DOI:** 10.1186/s13071-024-06509-w

**Published:** 2024-10-29

**Authors:** Trang Thuy Nguyen, Yudhi Ratna Nugraheni, Hoang Lan Anh Nguyen, Apinya Arnuphapprasert, Theerakamol Pengsakul, Le Quang Thong, Rinnara Ampol, Padet Siriyasatien, Morakot Kaewthamasorn

**Affiliations:** 1 The International Graduate Program of Veterinary Science and Technology (VST), Faculty of Veterinary Science, Chulalongkorn University, Bangkok, 10330 Thailand; 2 Department of Pathology, Faculty of Veterinary Science, Center of Excellence in Veterinary Parasitology, Chulalongkorn University, Bangkok, 10330 Thailand; 3 Department of Parasitology, Faculty of Veterinary Medicine, Universitas Gadjah Mada, Yogyakarta, 55281 Indonesia; 4 Faculty of Veterinary Medicine and Animal Husbandry, HUTECH University, Ho Chi Minh City, Vietnam; 5 Faculty of Veterinary Science, Rajamangala University of Technology Srivijaya, Nakhon Si Thammarat, 80240 Thailand; 6 Health and Environmental Research Center, Faculty of Environmental Management, Prince of Songkla University, Songkhla, 90110 Thailand; 7 Faculty of Animal Science and Veterinary Medicine, Nong Lam University, Ho Chi Minh City, Vietnam; 8 Department of Parasitology, Faculty of Medicine, Chulalongkorn University, Bangkok, 10330 Thailand

**Keywords:** Southern Vietnam, Sand flies, Mitochondrial cytochrome *b*, Cytochrome *c* oxidase subunit 1, PCR

## Abstract

**Background:**

Sand flies, belonging to the Psychodidae family, represent small, hairy insects that serve as significant vectors in various important medical and veterinary diseases. Despite being recognized by the World Health Organization as an endemic area for leishmaniasis, Southeast Asia lacks comprehensive information on the species composition and biology of sand flies. To address this, the current study aimed to survey sand fly biodiversity.

**Methods:**

Sand flies from six provinces in Southern Vietnam were collected using CDC light traps. Sand flies were subsequently identified morphologically and confirmed molecularly using mitochondrial cytochrome oxidase *c* subunit I (*COI*) and cytochrome *b* (*cytb*) sequences. BLASTN searches were conducted, and the species identity of sand flies was further confirmed through a Barcode of Life Database (BOLD) search utilizing *COI* sequences. Subsequently, nucleotide sequences were subjected to a panel of analyses including intraspecific variation, phylogenetic relationships and haplotype network. The average densities of collected sand flies (sand flies/trap/night) and species richness were also recorded.

**Results:**

A total of 753 sand flies were collected. After excluding damaged specimens, six sand fly species, namely *Phlebotomus stantoni*, *Sergentomyia khawi*, *Se. silvatica*, *Se. barraudi*, *Se. bailyi* and *Grassomyia indica*, were identified. All conspecific sand fly sequences, including *Ph. stantoni*, *Se. barraudi*, *Gr. indica*, *Se. bailyi*, *Se. khawi* and *Se. silvatica*, clustered with their reference sequences, corroborating the results of morphology-based identification, BLASTN analysis and BOLD search. For intraspecific variation of sand flies obtained from the current study, *COI* diversity indices were consistently higher than those of *cytb*.

**Conclusions:**

This study provides the first updates on morphological and molecular characterization of sand flies in Southern Vietnam. This acquired knowledge on sand fly species composition is essential for controlling sand fly-borne diseases in this potentially endemic region.

**Graphical Abstract:**

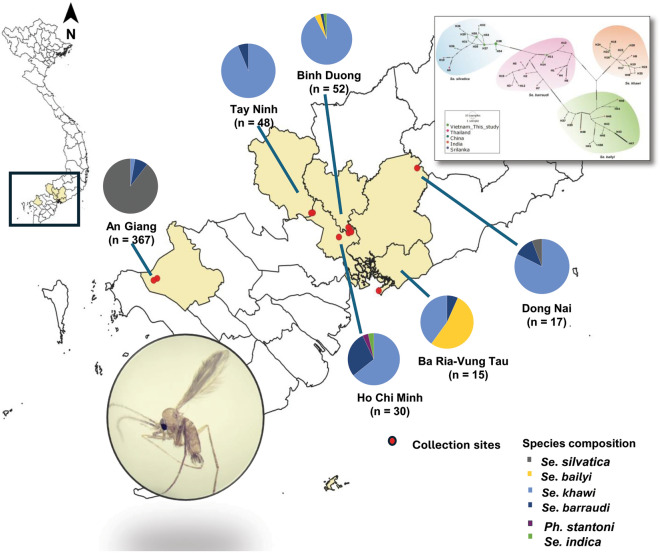

**Supplementary Information:**

The online version contains supplementary material available at 10.1186/s13071-024-06509-w.

## Background

Sand flies are small, hairy insects in the Diptera order, Psychodidae family and Phlebotominae subfamily. These hematophagous insects act as vectors for various disease agents such as *Phlebovirus* spp., *Bartonella* spp. and *Leishmania* spp. [1, 2]. Additionally, sand flies have been found to carry *Trypanosoma* DNA [3–6], but further experimental studies are needed to assess their potential involvement as vectors for trypanosomes. Sand fly-borne diseases are recognized as a global public health burden because of their ability to infect a wide range of hosts and cause severe consequences. Leishmaniasis, for instance, leads to an annual total of 20,000 to 30,000 deaths worldwide, with 0.9–1.6 million new cases reported each year, according to the Pan American Health Organization (PAHO) [7].

Despite Southeast Asia being recognized by the World Health Organization as an endemic area for sand fly-borne leishmaniasis [8], information regarding the vectorial competency and species distribution of this insect remains limited, except for Thailand, where extensive studies have been carried out on sand flies’ biology and sand fly-transmitted diseases. In Vietnam, a neighboring country with similar geographical characteristics and climatic conditions that are favorable to sand flies and the pathogens these insects carry, only a few studies have been conducted to investigate sand flies’ biology and the risk of sand fly-borne diseases. The most recent surveys on sand fly fauna in Vietnam were predominantly carried out in the northern part of the country, which has recorded 13 species of sand flies by morphology to date [9, 10]. In the southern part of the country, however, there has been no update on sand fly fauna since the 1930s [9], and pathogens in vectors and reservoir hosts are also virtually unexplored. This might result in underestimation or negligence regarding the disease’s burden, which in turn causes difficulties in prevention and control. Thus, further research on these pathogens is necessary to address the risk of sand fly-borne diseases in the country.

In this study, we aim to identify sand flies as potential vectors for several parasitic agents by assessing and comparing sand fly biodiversity in different regions of Southern Vietnam. This will contribute to improving monitoring and intervention strategies related to sand flies in the region.

## Methods

### Study sites and methods

**Fig. 1:**
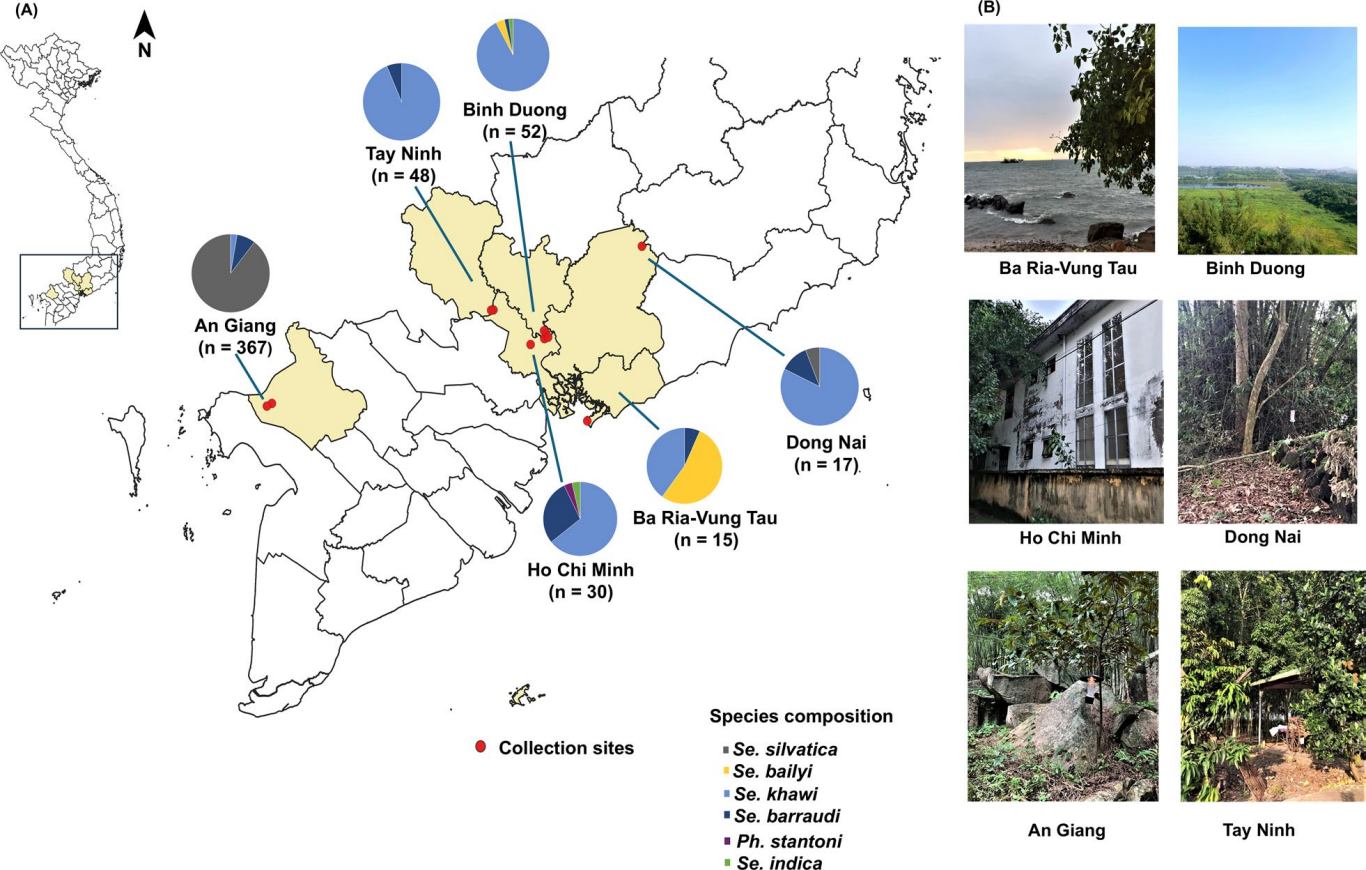
**A **Sample collection sites in six southern Vietnam provinces as well as the species composition and number of sand flies caught in these provinces are shown in the pie charts. **B **Photos were taken at locations where CDC light traps were placed, showing the surrounding areas

Six provinces in Southern Vietnam were selected to represent the various geographical characteristics and natural features of this region, comprising An Giang, Ho Chi Minh, Ba Ria-Vung Tau, Tay Ninh, Dong Nai and Binh Duong. The sampling sites are briefly described as follows: An Giang: Located in the southwestern region of Vietnam, An Giang is situated in the Mekong Delta between the Tien and Hau Rivers. The province is characterized by relatively flat terrain interwoven with a dense network of small rivers and canals. The western part of An Giang features mountainous areas, including the Cam Mountain range, which borders Cambodia. Sampling sites in this province were selected from rural residential areas and at the base of the mountains. Ho Chi Minh City: Situated in southeastern Vietnam, Ho Chi Minh City occupies flat terrain on the right bank of the Saigon River. The city shares borders with several provinces: Binh Duong to the north, Tay Ninh to the northwest, Dong Nai to the east and northeast, Ba Ria-Vung Tau to the southeast, Long An to the west and Tien Giang to the southwest. The city’s proximity to the Mekong Delta has led to the modification of tidal flats for agricultural purposes. Ultimately, this is intended to serve the study purpose of surveying sand flies’ fauna in different habitats in this part of the country, (Supplementary Table S1), which include forest, rock fissures/mountains and seaside as well as urban, suburban and rural farmhouses (Fig. 1). The annual rainfall averages ~ 2000 mm, with humidity levels ranging from 75 to 78% (https://climateknowledgeportal.worldbank.org/country/vietnam/climate-data-historical). Southern Vietnam has a tropical savanna climate, characterized by hot and humid conditions throughout the year. The region has two distinct seasons: a rainy season from May to November and a dry season from December to April. Temperatures consistently stay high, with an average of around 27 °C. Further detailed information regarding each collection site is provided in Supplementary Table S1.

### Sand fly collection and processing

Sand flies were collected using a convenience sampling method from May to July 2022, during the early rainy season, at 11 sites across six provinces. CDC miniature light traps (John W. Hock Co., Gainesville, FL, USA) were utilized to collect sand flies. Depending on the environmental conditions at each location, traps were placed either indoors or outdoors in areas known to harbor sand flies, such as animal sheds, areas near shrubs and trees, and rock fissures or cave entrances in forests or national parks. The traps were placed at a height of ~ 0.5–1.5 m above the ground and operated from 6:00 p.m. to 8:00 a.m. the following morning. In total, 29 light traps were deployed over 12 trap nights. At each site, 1–3 light traps were set, depending on the environmental conditions. Traps were placed either indoors or outdoors in areas known to harbor sand flies, such as animal sheds, areas near shrubs and bushes, and rock fissures or cave entrances in forests or national parks (Fig. 1).

After each trapping night, sand flies were carefully separated from other insect species. Only intact and well-preserved sandflies were chosen for further examination. Both male and female flies were sorted with total numbers recorded. Female sand flies were categorized based on their blood-feeding status, gravid or adult. The sorted sand flies were then transferred to 1.5 ml Eppendorf tubes containing 95–99.9% ethanol, and each tube was appropriately labeled with information regarding the trap, sex and capture date. For morphological identification, female sand flies were dissected on a glass slide in a drop of 1× PBS under a stereomicroscope. Taxonomic keys provided by Lewis [11] were utilized to guide the process. The dissected head and last three abdominal segments were mounted in Hoyer's medium, facilitating species identification through examination of the female's antenna, pharynx, cibarium and spermatheca. The remaining parts of the dissected sand flies were stored at − 20 °C for subsequent analysis.

### Molecular identification of sand flies

A NucleoSpin^®^ Tissue kit (Macherey–Nagel, Germany) was used to extract DNA from the dissected remains of sand flies, following the manufacturer's instructions. The extracted DNA was stored at − 20 °C for further analysis. To confirm the species of sand flies, representative individuals of each sand fly species from each province (identified by morphology) were selected for molecular identification and sequencing. These representatives were chosen from individuals that had been morphologically identified across all collection sites. Molecular identification was performed using conventional PCR (cPCR), targeting two molecular markers: mitochondrial cytochrome *b* (*cytb*) and cytochrome *c* oxidase subunit 1 (*COI*). The primers and protocols for amplifying these targets are detailed in Supplementary Table S2. PCR reactions were conducted in a final volume of 25 µl, comprising 4 µl DNA template, 0.2 µM final concentration of each primer, 0.02 U KOD Fx Neo polymerase (Toyobo, Japan), 0.4 mM of each dNTP and 1× PCR buffer for KOD Fx Neo. All PCR reactions were carried out using the Axygen^®^ MaxyGene Thermal Cycler (Life Science, USA). Visualization of PCR products was achieved through agarose gel electrophoresis, utilizing a 1.5% agarose gel stained with RedSafe™ (Intron Biotechnology, Korea) or ethidium bromide. The gel was visualized under a UV transilluminator.

### DNA sequencing

PCR-positive samples displaying clear, single bands were purified using ExoSAP-IT™ (Applied Biosystems, USA) per the manufacturer's instructions. For samples exhibiting multiple bands on gel electrophoresis, NucleoSpin^®^ Gel and PCR clean-up were employed for purification following the manufacturer's instructions. Subsequently, the purified DNA samples were submitted for bidirectional sequencing through U2Bio Co., Ltd. (https://www.u2bio.co.th/).

### Data analyses

For the collection of sand flies, the reported parameters included the total number of captured sand flies, classified by biological characteristics such as adult/blood-fed/gravid and male/female. Additional metrics comprised the average density of collected sand flies (sand flies/trap/night) and species richness (SR; number of species). The biological status of sand flies in the collection was outlined, providing details on the male-to-female ratio and female status (adult: blood-fed: gravid). Statistical analysis was performed using SPSS (v28) software. Descriptive statistics were utilized to analyze the distribution pattern and frequency of each sand fly species per province and collection habitat. Given the potential for small sample sizes (i.e. a low number of individuals per species at different locations), Fisher's exact test was applied to evaluate statistical significance (*P* < 0.05).

### Nucleotide sequence processing, estimates of intraspecific and interspecific sequence divergence, and phylogenetic inference

Raw nucleotide sequences obtained from Sanger sequencing were manually reviewed and edited using BioEdit software 7.0.5.3 [12] where necessary; low-quality sequences with ambiguous chromatographs were excluded from further analysis. Consequently, consensus sequences assembled from both forward and reverse directions were subjected to a BLASTn search in the GenBank™ database (https://blast.ncbi.nlm.nih.gov/Blast.cgi) to validate the study’s sequences against reference sequences. The sequences obtained from this study have been deposited in the GenBank^®^ database, as listed in Supplementary Table S3. *COI* sequences were also submitted to the Barcode of Life Database (BOLD), freely accessible at www.boldsystems.org, for further species verification. The evolutionary relationships of taxa using *COI* and *cytb* sequences were inferred with the neighbor-joining method. Evolutionary distances were calculated using the Kimura 2-Parameter method [13] implemented in MEGA software, version 11.0.13 [14]. Estimates of evolutionary divergence between *COI* and *cytb* sequences for intra- and interspecific variation were conducted using MEGA software [14]. The analyses were performed using the Kimura 2-Parameter model [13]. One to two conspecific sequences from each sampling location in each province were chosen as representatives for constructing the phylogenetic trees. Additionally, we prioritized specimens that had both *COI* and *cytb* sequences to allow referencing with both markers. Haplotypes were also considered when selecting specimens for inclusion in the phylogenetic analysis; we aimed to include unique haplotypes from each province in the phylogenetic tree (unique haplotypes included in the phylogenetic analysis are bolded; for *COI*, see Supplementary Table S4: *COI* haplotypes of sand flies used to construct the TCS network). The intraspecific relationships of taxa were also illustrated in the haplotype network, with all unique haplotypes included. For the additional *cytb* tree, similar criteria were applied when selecting sequences for phylogenetic analysis. Phylogenetic trees were generated with IQTREE 1.6.12 (http://www.iqtree.org/) [15] using the maximum likelihood method (1000 bootstraps). The Bayesian inference method was executed using MrBayes 3.2.7 (10,000,000 MCMC) on CIPRES Science Gateway. The best-fit model for the ML tree of the *COI* gene was GTR + F + I + G4, while the best-fit model for the ML tree of the *cytb* gene was HKY + F + G4. The tree file was visualized and edited in the iTOL version 6.8.1 (Interactive Tree Of Life; http://www.itol.embl.de) [16]. For genetic diversity analysis, sequences were assessed using DnaSP software (available at www.ub.edu/dnasp). Haplotype networks were visualized using the minimum spanning method in Population Analysis with Reticulate Trees (PopART) [17].

## Results

### Sand fly collection

In total, 753 sand flies were collected from six provinces in Southern Vietnam. Of these, 529 specimens were females, dominating the collection and doubling the count of male sand flies (224 specimens). Regarding female sand flies, An Giang province exhibited the highest number of collected specimens (367 specimens), followed by Binh Duong (52 specimens), Tay Ninh (48 specimens), Ho Chi Minh (30 specimens), Dong Nai (17 specimens) and Ba Ria Vung Tau (15 specimens). The paramount instance of females per trap was documented in An Giang, registering 141 specimens in June 2022. Gravid females were documented in 213 specimens. Notably, none of the female sand flies in the collected samples exhibited visible blood meals in the abdomen.

Morphological identification of 529 female sand flies revealed their classification into six species and three genera, namely *Phlebotomus, Sergentomyia* and *Grassomyia*, with *Sergentomyia* emerging as the predominant genus. The identified species included *Sergentomyia (Se.) silvatica* (64.1%, *n* = 339), *Se. khawi* (24.4%, *n* = 129), *Se. barraudi* (9.1%, *n* = 48), *Phlebotomus (Ph.) stantoni* (0.4%, *n* = 2), *Se. bailyi* (1.9%, *n* = 10) and *Grassomyia (Gr.) indica* (0.2%, *n* = 1) (Supplementary Fig. S1). The species richness across provinces was delineated in descending order: Ho Chi Minh (Ho Chi Minh = 4), Binh Duong (Binh Duong = 3), Dong Nai (Dong Nai = 3), Ba Ria-Vung Tau (Ba Ria-Vung Tau = 3), An Giang (An Giang = 3) and Tay Ninh (Tay Ninh = 2). Notably, in this collection, *Se. barraudi* and *Se. khawi* were ubiquitous across all provinces, while *Ph. stantoni* was only found in Ho Chi Minh. Within Southeastern Vietnam (Dong Nai, Ho Chi Minh, Binh Duong, Tay Ninh), *Se. khawi* emerged as the predominant species, whereas *Se. silvatica* claimed the highest abundance in the Southwest province of An Giang. The intricacies of sand fly fauna across the six provinces of Southern Vietnam are visually represented in Fig. 1.

Additionally, regarding habitat, the disparity in sand fly captures was found to be statistically significant (*P* < 0.001). The mountainous/cave environments emerged as the most prolific, hosting the highest abundance of sand flies in this study. Species-wise, *Se. silvatica* dominated the habitat in the southwestern region of Vietnam. Across diverse habitats, *Se. barraudi* and *Se. khawi* exhibited wide distribution, with *Se. barraudi* displaying ubiquity across all environments. Detailed summaries of sand fly numbers, categorized by habitats and species, are provided in Table 1.

**Table 1:** Number of sand flies captured by habitat in Vietnam

Species	Habitat						
	Urban	Suburb	Rural farmhouse	Seaside	Forest	Rock fissure/moutain	Total (by species)
*Ph. stantoni*	2	0	0	0	0	0	2
*Se. bailyi*	0	2	0	8	0	0	10
*Se. barraudi*^c^	2	9	22	6	2	7	48
*Se. silvatica*^a^	0	0	94	0	1	244	339
*Se. khawi*^b^^,c^	0	65	48	1	13	2	129
*Grassomyia indica*	0	1	0	0	0	0	1
Total (by habitat)	4	77	164	15	16	253	529

^a^Predominant species found in Southwestern Vietnam

^b^Predominant species found in Southeastern Vietnam

^c^Species found in most habitats

### Molecular analysis of sand flies

A total of 84 sequences (48 *COI* and 36 *cytb*)encompassing six sand fly species from Southern Vietnam were successfully obtained through the sequencing of 48 representative individual specimens from each sampling location (GenBank accession number of analyzed sequences provided in Supplementary Table S3). Subsequent BLASTN analysis was conducted across all *COI* and *cytb* sequences, and the species identity of sand flies by *COI* was further confirmed through a BOLD search of *COI* sequences. The outcomes of BLASTN and BOLD searches are succinctly presented in Table 2. Sequences exhibiting a high percentage of identity (≥ 97%) with references previously deposited in GenBank or BOLD databases were assigned to the corresponding species. The designated species ID was established based on consistency among search results of two genetic markers, alignment with morphological identification and its placement in the phylogenetic tree. Resolution of the conflicting findings was also achieved through consultation with previous taxonomic literature.

**Table 2:** Morphological identification and nucleotide search results of sand flies collected from this study

Morphological-based ID	BOLD Search			GenBank Search						Designated ID from sequence searches
	*COI* gene			*COI* gene			*cytb* gene			
	Closest sequence	Average identity (%)	No. of sequences	Closest sequence	Average identity (%)		Closest sequence	Average identity (%)	No. of sequences	
*Ph. stantoni*	JSTH035–16 *Ph. stantoni*	99.62	1	LC136898 *Ph. stantoni*	99.61		*–*	–		*Ph. stantoni*
*Se. barraudi*	LC136903 *Se. barraudi*	99.91	7	LC136903 *Se. barraudi*	99.92		MG770902 *Se. barraudi*	99.42	6	*Se. barraudi*
*Se. bailyi*	MN850825 *Se. bailyi*	99.19	4	MN850825 *Se. bailyi*	98.92		MN853013 *Se. bailyi*	99.34	4	*Se. bailyi*
*Se. khawi*	LC136894 (100%) *Se. gemmea* MN850819 (100%) *Se. khawi*	99.93 98.45	13	MZ400961 *Se. gemmea*	99.56		JX852706 *Se. gemmea* (100%) MK460569 *Se. khawi (100%)* OK398070 *Se. khawi*	99.81	6	*Se. khawi*
*Se. silvatica*	JSDB005 *Se. silvatica*	95.52	23	OP879807 *Se. sylvatica*	95.03		EU143782 *Se. anka*	90. 8	20	*Se. silvatica*

Most of the sand fly species collected in this study, including *Ph. stantoni, Se. barraudi* and *Se. bailyi*, as initially identified based on morphology, demonstrated high similarity with conspecific sequences of *COI* and *cytb* markers with average percent similarity > 98% in both BOLD and GenBank databases. With *Se. khawi*, on the other hand, conflicting search results of both genetic markers were noticeable in both BOLD and GenBank databases. The designated IDs for *Se. khawi* were ultimately made based on several pieces of evidence including the morphological identification and BOLD and BLASTN search results, which were closely matched with either *Se. khawi* or *Sergentomyia gemmea*, and consulting relevant literature.

For *Se. silvatica*, BOLD and BLASTN searches utilizing *COI* sequences indicated an average of 95.52% identity to *Se. silvatica* (BOLD accession number: JSDB005) and 95.03% identity to *Se. silvatica* (GenBank accession number: OP879807). Conversely, *Cytochrome b* (*cytb*) BLASTN search showed a relatively lower percent identity (90.8%) with the Madagascan *Sergentomyia anka* (EU143872). Therefore, the final designated ID for *Se. silvatica* was assigned based on the cumulative evidence from both BOLD and BLASTN searches, accompanied by morphological identifications. *Grassomyia indica* sequences were excluded from this analysis because of ambiguous chromatograms.

### Phylogenetic analysis of sand fly COI and cytb genes

**Fig. 2:**
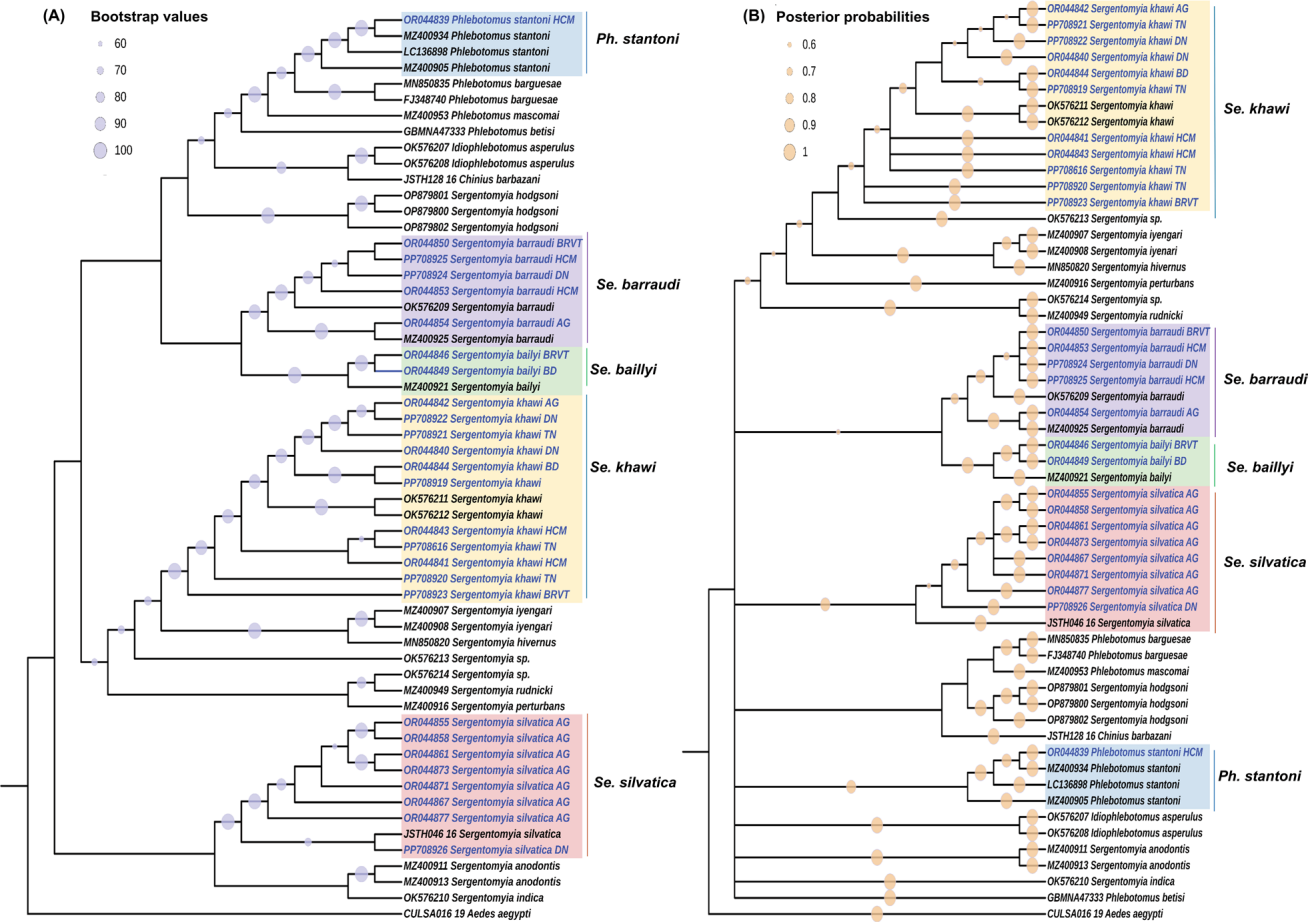
**A** Maximum likelihood (ML) and (**B**) Bayesian interference (BI) phylogenetic tree illustrates *COI* sequences (539 bp) of sand flies from this study alongside references from GenBank and BOLD databases. ML bootstrap values > 60 and BI posterior probabilities > 0.6 are shown at nodes. The ML analysis involved 1000 replications, while the BI analysis incorporated 10,000,000 MCMC iterations. Sequences originating from this study are colored blue. The following letter codes represent the sampling sites: AG: An Giang, DN: Dong Nai, HCM: Ho Chi Minh, TN: Tay Ninh, BD: Binh Duong, BRVT: Ba Ria-Vung Tau

For phylogenetic analysis of both *COI* and *cytb*, one to two conspecific sequences from each sampling location at every province were selected to be included in the tree construction. A total of 57 *COI* sequences were incorporated into the construction of maximum likelihood (ML) (Fig. 2a) and Bayesian inference (BI) (Fig. 2b) phylogenetic trees, encompassing 27 sequences from the present study and 30 *COI* references from GenBank and BOLD databases. The *Aedes aegypti COI* sequences from the BOLD database (BOLD ID: CULSA016-19) were employed as an outgroup.

**Fig. 3:**
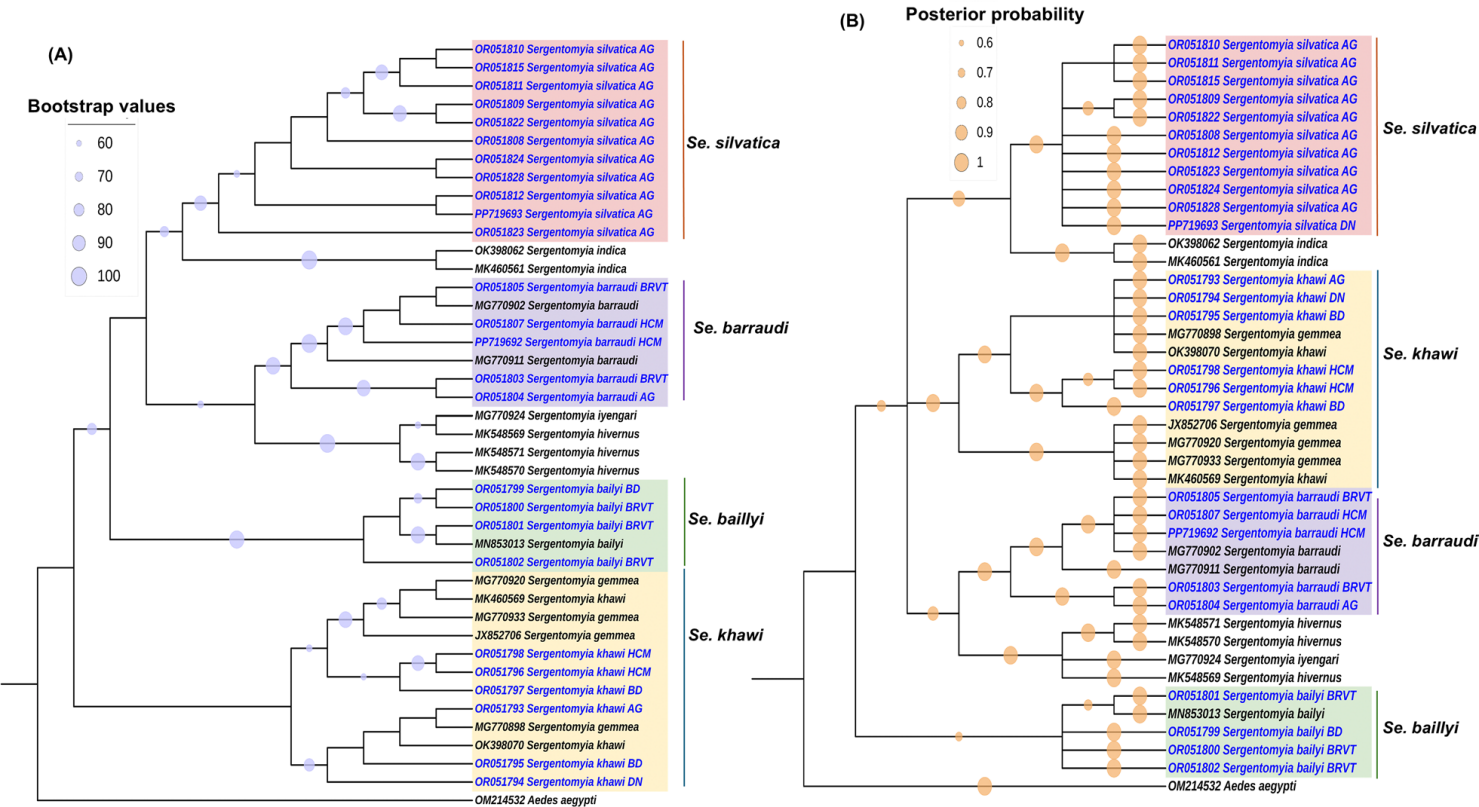
**A** Maximum likelihood (ML) cladogram and (**B**) Bayesian inference (BI) cladogram represent sand fly *cytochrome b (cytb*) sequences from this study, comprising 380 base pairs, in conjunction with references from GenBank and BOLD databases. ML bootstrap values > 60 and BI posterior probabilities > 0.6 are shown at nodes. The ML analysis involved 1000 replications, while the BI analysis incorporated 10,000,000 MCMC iterations. Sequences originating from this study are colored blue. The following letter codes represent the sampling sites: AG: An Giang, DN: Dong Nai, HCM: Ho Chi Minh, TN: Tay Ninh, BD: Binh Duong, BRVT: Ba Ria-Vung Tau

While there was a slight discrepancy in the order of species between ML and BI trees, all conspecific sand fly sequences, comprising *Ph. stantoni, Se. barraudi, Gr. indica, Se. bailyi*, *Se. khawi* and *Se. sylvatica*, were clustered with their reference sequences, corroborating the results of morphology-based identification, BLASTN analysis and BOLD search. Furthermore, ML and BI phylogenetic trees of sand fly *cytb* were also constructed using a total of 42 sequences, comprising 26 sequences successfully obtained from this study and 16 references from the GenBank database. The *Aedes aegypti cytb* sequence was used as an outgroup (accession no.: OM214532) (Fig. 3a, b). The ML and BI phylogenetic trees inferred from *cytb* sequences were consistent with ML and BI trees utilizing *COI* sequences for all species. Conspecific sequences identified from this study were found clustering with their respective reference sequences. Notably, in the *Se. khawi* clade, *cytb* sequences from this study were positioned alongside both *Se. gemmea* and *Se. khawi* references originating from different regions of Thailand, suggesting potential synonymy between the referenced *Se. gemmea* and *Se. khawi*.

### Genetic variations and estimates of intra- and interspecific sequence divergences of COI and cytb sequences in dominant sand fly species from Southern Vietnam

The *COI* and *cytb* sequences of sand flies from four main species in Southern Vietnam were subjected to genetic diversity analysis, focusing on individually processed specimens to avoid potential misinterpretations (Table 3). The haplotype diversity (Hd) for *COI* was generally high across all species, ranging from 0.86 (*Se. barraudi*) to 1 (*Se. bailyi*). Meanwhile, nucleotide diversity (π) remained relatively low for all species, varying between 0.007 (*Se. silvatica*) and 0.043 (*Se. barraudi*) in Southern Vietnam.

**Table 3:** Genetic diversity indices and neutrality test of *COI* and *cytb* sequences from four species of sand flies in Southern Vietnam

Species	Marker	*N*	Size (bp)	*S*	*h*	Hd ± SD	π ± SD	Tajima’s D	Fu and Li’s D
*Se. khawi*	*COI*	13	651	17	11	0.974 ± 0.04	0.009 ± 0.001	− 0.16	− 0.66
	*cytb*	6	487	12	4	0.8 ± 0.17	0.011 ± 0.002	0.25	0.08
*Se. silvatica*	*COI*	23	654	27	14	0.92 ± 0.04	0.007 ± 0.002	− 1.34	− 2.03
	***cytb***	**20**	**488**	**15**	**10**	**0.85 ± 0.07**	**0.004 ± 0.001**	− 1.96*	− 2.66*
*Se. barraudi*	*COI*	7	654	66	5	0.86 ± 0.14	0.043 ± 0.015	0.07	0.56
	*cytb*	6	495	41	3	0.6 ± 0.05	0.042 ± 0.014	0.81	1.11
*Se. bailyi*	*COI*	4	654	17	4	1 ± 0.18	0.013 ± 0.003	− 0.85	− 0.85
	*cytb*	4	495	6	4	1 ± 0.18	0.006 ± 0.002	− 0.81	− 0.81

*N* number of analyzed sequences, *S* number of polymorphic sites, *h* number of haplotypes, *Hd* haplotype diversity, *π* nucleotide diversity, *SD* standard deviation

*Statistically significant test results of Tajima’s D and Fu and Li’s D (*P* < 0.05)

For *cytb*, the Hd range was broader, with the upper limit sustained at 1 in *Se. bailyi* and the lower range changed to 0.667 in *Se. silvatica*. Nucleotide diversity remained low for all species, with the *cytb* nucleotide diversity range being lower than that of *COI*. Notably, within the same species, *COI* diversity indices were consistently higher than those of *cytb*. This observation suggests that, for the sequences of analyzed species, *COI* is the more genetically diverse gene between the two markers. However, it is acknowledged that this discrepancy might result from the lower number of *cytb* sequences and shorter sequence length analyzed (487–495 bp) compared to *COI* (654 bp).

Neutrality tests using Tajima’s D and Fu’s and Li’s D* were not statistically significant (*p* > 0.1) for *COI* in all sand fly species tested. This suggests that, genetically, the population of the tested sand fly species (at the time of sample collection) was in neutral equilibrium. However, the low number of sequences in the analysis might have contributed to these non-significant results. Similar results were observed in the analysis of *cytb* in most species, except for *Se. sylvatica*, where neutrality tests were negative and statistically significant (*P* < 0.05), implying a recent population expansion or selection sweep after a bottleneck at the gene locus tested [18].

Intraspecific sequence divergence of the *COI* gene among dominant sand fly species was < 0.027, except for *Se. barraudi* (accession no. OR044854), which was relatively higher than that of the other species, ranging from 0.098 to 0.100 (Table 4). Intraspecific sequence divergence of the *cytb* gene was ≤ 0.019, which was slightly lower than what was observed in the *COI* gene, except among populations of *Se. barraudi*, where intraspecific sequence divergence was relatively higher than in the other species assessed (ranging from 0.068 to 0.078) (Table 5). Interspecific sequence divergence of the *COI* gene among dominant sand fly species ranged from 0.132 to 0.185, which was slightly higher than that of the *cytb* gene (0.105–0.164). In addition to estimates of intra- and interspecific sequence divergence, the evolutionary relationships of taxa obtained in the present study, inferred using the neighbor-joining method, are provided in Supplementary Figs. S2 and S3.

**Table 4:**
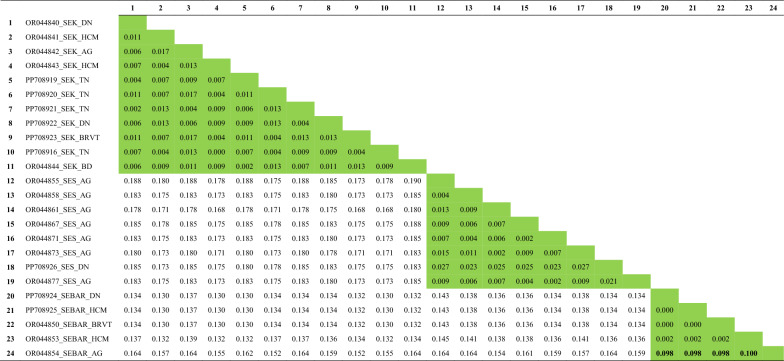
Estimates of intra- and interspecific sequence divergences as measured by the *COI* gene

Intraspecific sequence divergences are shaded in green, while interspecific sequence divergences are unshaded. The sequences (Nos. 1–24) are arranged by GenBank accession number, followed by the initial of the species name and the initial of the sampling site origin. The full species names are as follows: SEK, *Se. khawi*; SES, *Se. silvatica*; SEBAR, *Se. barraudi.* It should be noted that intraspecific sequence divergences among *Se. barraudi* (numbers in bold) are relatively high compared to the other species

**Table 5:**
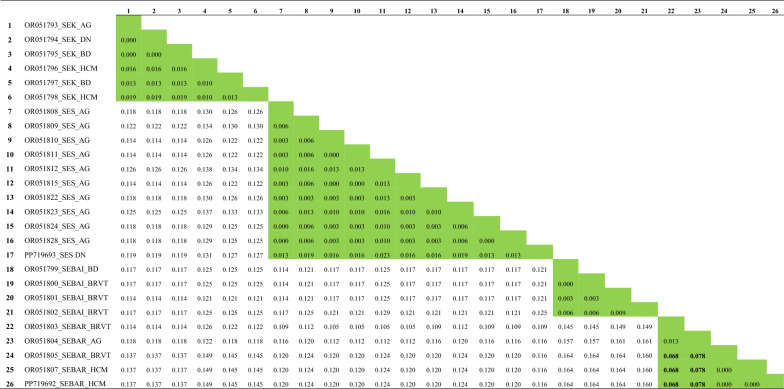
Estimates of intra- and interspecific sequence divergences as measured by the *cytb* gene

Intraspecific sequence divergences are shaded in green, while interspecific sequence divergences are unshaded. The sequences (Nos. 1–26) are arranged by GenBank accession number, followed by the initial of the species name and the initial of the sampling site origin. The full species names are as follows: SEK, *Se. khawi*; SES, *Se. silvatica*; SEBAI, *Se. bailyi;* SEBAR, *Se. barraudi.* It should be noted that intraspecific sequence divergences among *Se. barraudi* (numbers in bold) are relatively high compared to the other species

### Haplotype network analysis based on the COI sequences in dominant species of sand flies present in Southern Vietnam

**Fig. 4:**
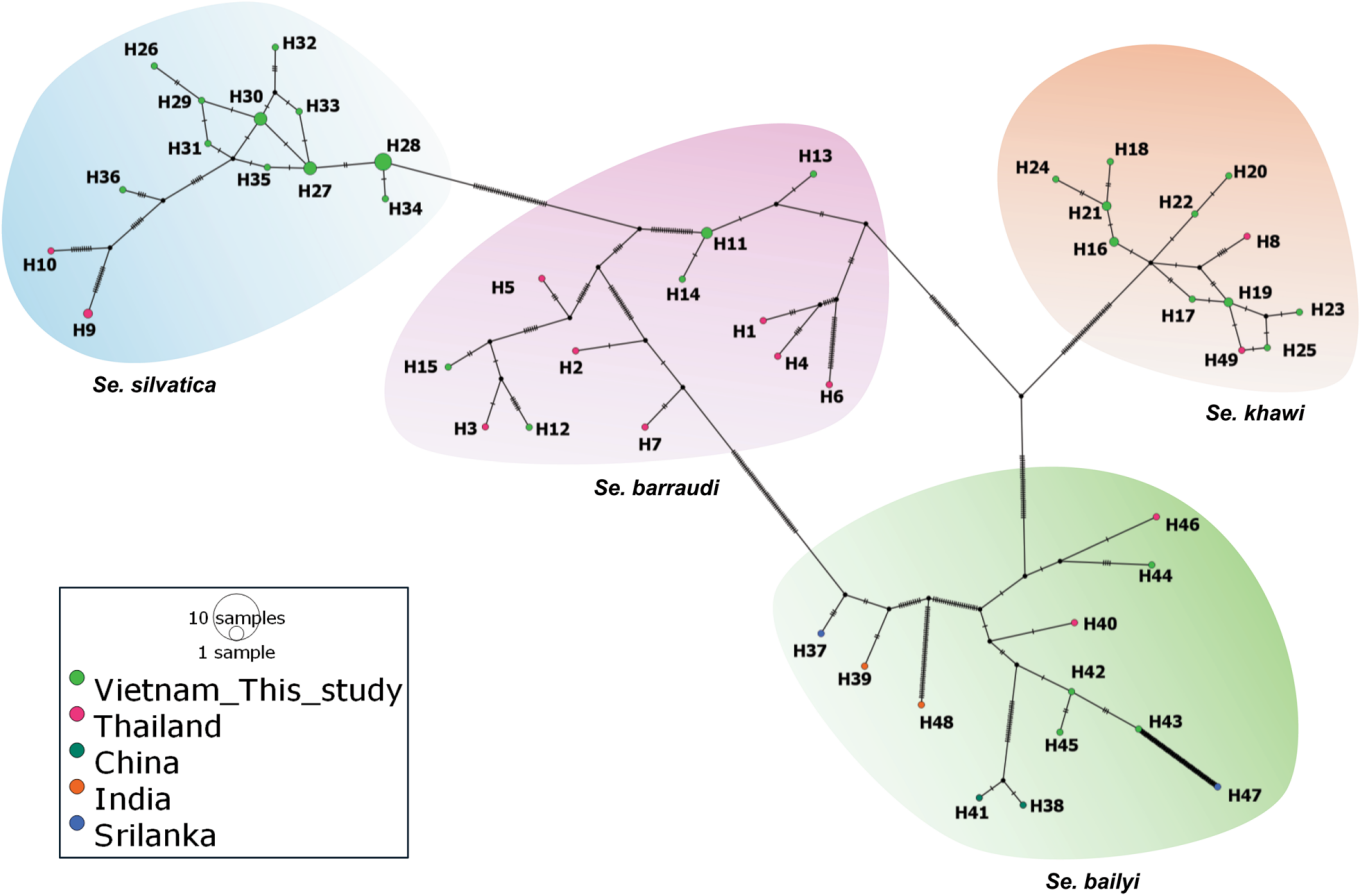
The TCS haplotype network depicts the *COI* sequences of sand flies from four species found in Southern Vietnam, encompassing a sequence length of 477 bp. Each circle within the network corresponds to a unique haplotype, with circle size proportionate to the frequency of each sequence within the haplotype. Nucleotide polymorphisms between haplotypes are denoted by hatch marks along the connecting lines. The figure employs orange, pink, green and blue shadows to demarcate four distinct clusters representing *Se. khawi, Se. barraudi, Se. bailyi* and *Se. silvatica*. These clusters provide a species-specific delineation, facilitating visual comprehension of the genetic relationships among haplotypes within and between sand fly species

A TCS haplotype network was constructed using 67 *COI* sequences (477 bp) obtained from Southern Vietnam and GenBank references, encompassing 26 *Se. silvatica* sequences, 15 *Se. khawi* sequences, 14 sequences from *Se. barraudi* and 12 *Se. bailyi* sequences (Fig. 4).

The TCS haplotype network reaffirmed the findings of the genetic diversity analysis. Within this set of *COI* sequences from Southern Vietnam, 49 haplotypes (H) were categorized into four distinct clusters by species. *Sergentomyia barraudi*, *Se. khawi*, *Se. silvatica* and *Se. bailyi* exhibited 12, 12, 13 and 12 haplotypes, respectively (Supplementary Table S4). Within each species cluster, haplotypes from Vietnam (symbolized by bright green circles) were distributed and grouped distinctively. In the *Se. khawi* and *Se. silvatica* clusters, most Vietnam haplotypes exhibited low haplotype diversity, differing by one to three nucleotides from the nearest haplotype.

In the *Se. barraudi* and *Se. bailyi* clusters, Vietnam haplotypes were more sparsely distributed. With *Se. barraudi*, among the haplotypes from Vietnam, the group of H11, H13 and H14 clustered together, distant from the other two haplotypes (H12, H15). Similarly, three haplotypes in the *Se. bailyi* group (H43, H44, H45) formed a distinct group, while H45 linked more closely to the Thai references (H46). In every species cluster, Thailand haplotypes (pink circles) were observed to share a close relationship with *COI* haplotypes from Southern Vietnam. Within the *Se. bailyi* cluster, haplotypes from Sri Lanka (blue circle) and India (orange circle) were distant from their counterparts originating from both the same and different countries. The sand flies' *cytb* haplotype network of these four species was not analyzed because of the lack of relevant reference sequences deposited in databases.

## Discussion

In Vietnam, limited research has been done on both sand flies and sand fly-borne pathogens, leading to a lack of updates on vectors and disease information and ultimately negligence regarding the disease’s burden. In 2020, Vu et al. [9] published the group’s update on sand fly species in Quang Ninh province (northern Vietnam), which were the first internationally available data on sand fly systematics since the 1930s. Solely based on morphological identification of both males and females, 10 species of sand flies of 4 genera were recorded, with *Sergentomyia* being predominant (79.7%), followed by the *Phlebotomus* genus (13.7%), *Chinius* genus (6.1%) and *Idiophlebotomus* genus (0.8%), respectively. The 10 species were reported as *Se. silvatica*, *Se. barraudi*, *Sergentomyia hivernus*, *Se. bailyi*, *Phlebotomus mascomai*, *Ph. stantoni*, *Ph. yunshengensis*, *Ph. betisi*, *Chinius (Ch.) junlianensis* and *Idiophlebotomus (Id.) longiforceps*. In 2021, this group of researchers conducted larger scale research on sand flies in six northern provinces of Vietnam, confirming the results from their previous study that the genus *Sergentomyia* prevailed over other genera. In the six provinces studied, five genera and 13 species were found, in which the three most abundant species were *Se. barraudi*, *Se. silvatica* and *Ph. stantoni* [10].

As previously outlined, the current investigation identified a total of six sand fly species across six provinces in Southern Vietnam, namely *Ph. stantoni, Se. barraudi, Se. silvatica, Se. khawi, Se. bailyi* and *Gr. indica*. These species have historical records in various regions of Vietnam, spanning the south central, northern and southwestern regions, with early documentation dating back to as early as the 1930s [11, 19–23]. These results also coincide with the study of Vu et al. [9, 10], where these species were also identified in Northern Vietnam as part of a 13-species sand fly collection. Moreover, these six species have also been reported in China, Laos, Cambodia, Malaysia and Thailand [11, 22–26]. These results substantiate the widespread distribution of these six species not only in Northern and Southern Vietnam but also in neighboring countries.

Based on observation by Hustedt et al. [26], in the Greater Mekong region, the reported sand fly species richness per country reflected the number of entomological studies conducted. As a result, according to the review from the same research group [26], compared with Thailand (SR = 34) and Malaysia (SR = 32), sand fly fauna in Vietnam were less abundant (SR = 18). Within Vietnam, compared with recent studies from Northern Vietnam, the richness of sand fly species in Northern Vietnam (SR = 13) exceeded that in Southern Vietnam (SR = 6). Beside the lesser number of entomological updates as stated above, this disparity may also be attributed to the more extensive period of sample collection and potential habitat and environmental influences. Additionally, the larger number of traps employed in the studies of Vu et al. [9, 10] affected the number of sand flies captured in different locations and, therefore, possibly had an impact on the species richness of sand flies. The collection method may also influence the number of sand flies captured and the species richness, according to Alexander [27]. Future entomological studies in Southern Vietnam should be conducted systematically on a larger scale, with strategic sampling timing and the utilization of traps. In this study, sand flies were captured in various habitats, revealing a correlation between habitat and the number of sand flies caught (*P* < 0.05). The mountainous/rock fissure habitat harbored the highest number of sand flies, aligning with research by Vu et al. [9]. This can be attributed to the characteristic structures of rocky crevices and ridges that sand flies favor [27, 28].

The activities of sand flies follow a seasonal and nocturnal/diurnal pattern that differs between regions. For example, Jaturas et al. [29] reported that the sand flies’ peak intervals in Phitsanulok, Northern Thailand, were from 00:01–02:00 and 22:01–00:00, with May and July being seasonal peaks, while the flies’ seasonal peaks were late April and mid-September in Iran [30]. These characteristics should be considered in experimental design for effective sample collection and correct methodologies in studies about disease dynamics. Unfortunately, the seasonal variation of sand fly species could not be explored in this study, as most sand flies were collected during the rainy seasons. Future studies should consider analyzing seasonal variation as a factor in determining true diversity and aiding in vector incrimination. Additionally, only female sand flies were analyzed in this study, which is justified given their role in disease transmission. However, for a systematic and comprehensive understanding of sand fly diversity in a particular location, the speciation of male sand flies should be considered.

Traditionally, the identification of sand fly species relies on the observation of morphological features such as cibarium teeth, spermatheca, pharynx and antennal ascoidal spurs, each unique to specific sand fly species. This method, while generally effective, demands considerable expertise and experience. Challenges arise from cryptic species complexes and subtle morphological differences, leading to instances of misidentification, as reported in various studies. Phuphisut et al. [24] documented misidentifications of *Sergentomyia gemmea* as *Se. iyengari* and vice versa, while Preativatanyou et al. [31] highlighted the ambiguity between *Se. gemmea* and *Se. khawi.* Moreover, historical records of *Se. iyengari* in Southeast Asia may refer to *Se. khawi*, according to Vu et al. [10]. The synonymization of *Se. iyengari* with *Se. hivernus* has a more complicated taxonomy [32]. This implies the necessity for extensive and standardized morphological keys for the classification of sand fly species in Southeast Asian countries. The development and application of more advanced techniques, especially molecular methods, are also vital for precise species identification.

Relying heavily on previous key identification literature also raises the risk of overlooking new species, undermining the true species diversity of sand flies in a studied area. In Thailand, recent examinations of morphological features have led to the reporting of new sand fly species [33], prompting the need for molecular methods to enhance identification accuracy. Researchers advocate the integrative use of molecular methods with classic morphology identification, acknowledging that relying solely on molecular methods can be misleading, especially for related species with nuanced differences and when only 37% of nominal sand fly species have been molecularly characterized [34]. For DNA-based methods, the use of multiple genetic markers, preferably with different rates of evolution, has been proposed to enhance the credibility of identification [34, 35]. Mitochondrial *COI* and *cytb* are widely used markers considered suitable for species identification [36].

In the current study, sand flies underwent initial morphological identification, and the results were molecularly confirmed by querying consensus sequences of *COI* and *cytb* on GenBank and BOLD (*COI*) databases and through phylogenetic analysis of *COI* and *cytb* sequences. While the preliminary results from BLASTN and BOLD queries were inconclusive for certain species not thoroughly molecularly characterized, phylogenetic analysis clarified the identity of almost all morphospecies in the current study with high BI posterior probability and ML bootstrap supports. This integrative approach proved suitable for the identification of sand flies in Southern Vietnam, with results complementing one another and supporting species identification via morphology.

In this analysis, we delved into the intra- and interspecific variability of two molecular markers, *COI* and *cytb*, to unravel the population structure and genetic divergence of sand flies. Among the five species collected and analyzed in Southern Vietnam, excluding *Se. barraudi*'s *cytb*, both *COI* and *cytb* exhibited high haplotype diversity, while nucleotide diversity values were relatively low. This suggests that haplotypes were closely related within each sand fly population, differing by only one or two nucleotides. This pattern is often associated with population expansion following a period of reduced population size, as proposed by Grant and Bowen [37]. The genetic diversity results were visualized in haplotype networks for the four species, *Se. bailyi*, *Se. barraudi*, *Se. khawi* and *Se. silvatica*. Haplotypes from the same species clustered together, consistent with previous findings. The haplotype network analysis suggests the potential role of geographical distance in the genetic divergence of sand flies' mitochondrial genes, as haplotypes from the same country or with closer geographical distance origin shared stronger relationships. This is evidenced by the grouping of Southern Vietnam haplotypes with one another, as well as the clustering of the Vietnam and Thai haplotypes in *Se. bailyi* species. Within the same country and the same species, haplotypes from southeastern provinces are positioned further from southwestern ones, as exemplified by *Se. silvatica*’s An Giang (Southwestern) haplotype (H36) and the rest of the haplotypes (Southeastern origin). This geography-driven genetic differentiation was previously quantified by Lee and Mitchell-Olds [38]. The aforementioned study also concluded that the combination of geographical and environmental factors produced more distinct genetic divergence compared to distance alone. This could be the explanation for the isolation of Sri Lanka and Indian haplotypes from the rest of Southern China, Thailand and Southern Vietnam in *Se. bailyi* from this study. Notably, our current estimates of intraspecific sequence divergence in sand flies from Southern Vietnam (particularly *Se. barraudi* and *Se. baillyi*) show significant nucleotide differences in the *COI* gene compared to other species, which is consistent with the results of the haplotype network analysis. This may suggest potential cryptic speciation or misidentification of the specimens, warranting further study. Overall, this analysis provides fundamental insights into the genetic diversity of sand flies in Southern Vietnam based on the *COI* and *cytb* genes. The results underscore a high level of intraspecific diversity that does not necessarily translate into substantial haplotypic differences. As sample size and sequence length can significantly impact analysis outcomes, future studies should carefully consider an adequate number of sequences for each species.

Regarding the possible link between the sand fly fauna discovered in this study and sand fly-borne diseases, previously in Thailand, *Leishmania* DNA was detected in *Gr. indica* and *Ph. stantoni* by Preativatanyou et al. [31] and *Se. khawi* by Srisuton et al. [5]. For *Trypanosoma*, the parasites’ DNA was also found in various sand fly species from Thailand, including *Ph. stantoni*, *Idiophlebotomus asperulus*, *Se. khawi*, *Se. barraudi* and *Phlebotomus betisi*, with varied infection rates. Furthermore, living anuran *Trypanosoma* was microscopically detected in *Gr. indica* [31]. Consequently, these species were considered potential vectors of *Trypanosoma* in this country. Accordingly, *Ph. stantoni*, *Se. khawi*, *Se. barraudi* and *Gr. indica* were found to be present in Vietnam, more specifically in Southern Vietnam, as reported in this study, indicating the possible role these species might play in sand fly-borne disease transmission in this country. This speculation aligns with the findings from Vu et al. [9, 10], in which *Ph. stantoni* was captured in the house of a previously reported leishmaniasis patient from Northern Vietnam. However, further investigations are required to assess the sand fly-borne disease risk as well as the potential role these insects play in the transmission cycle in Vietnam.

## Conclusions

This study provides the first comprehensive update on sand fly taxonomy in Southern Vietnam since the 1930s and also offers the pioneering molecular characterization of sand flies by *COI* and *cytb* in the country. Ultimately, this understanding will contribute significantly to the development and enhancement of disease surveillance, control and prevention programs in the researched regions. For future study, the importance of further research on the vector competence, host preference, seasonality and biology of sand flies should be emphasized.

## Supplementary Information

Additional file 1: Supplementary Figure S1. The morphological characteristics of sand fly species identified in this study are as follows: a pair of cibarium and spermatheca of *Sergentomyia bailyi* (Aa), *Se. barraudi* (Bb), *Se. silvatica* (Cc), *Se. khawi* (Dd), *Phlebotomus stantoni* (Ee), *Grassomyia indica* (Ff). Black arrow: cibarium; arrowhead: spermatheca.

Additional file 2: Supplementary Figure S2. The evolutionary relationships of taxa based on *COI* sequences were inferred using the neighbor-joining method. The percentage of replicate trees in which the associated taxa clustered together during the bootstrap test (1000 replicates) is shown above the branches. The evolutionary distances were calculated using the Kimura 2-Parameter method [13] and are expressed as the number of base substitutions per site. The following letter codes represent the sampling sites: AG: An Giang, DN: Dong Nai, HCM: Ho Chi Minh, TN: Tay Ninh, BD: Binh Duong, BRVT: Ba Ria-Vung Tau.

Additional file 3: Supplementary Figure S3. The evolutionary relationships of taxa based on *cytb* sequences were inferred using the neighbor-joining method. The percentage of replicate trees in which the associated taxa clustered together during the bootstrap test (1000 replicates) is shown above the branches. The evolutionary distances were calculated using the Kimura 2-Parameter method [13] and are expressed as the number of base substitutions per site. The following letter codes represent the sampling sites: AG: An Giang, DN: Dong Nai, HCM: Ho Chi Minh, TN: Tay Ninh, BD: Binh Duong, BRVT: Ba Ria-Vung Tau.

Additional file 4: Supplementary Table S1. Additional details of the collection sites, including information on vegetation, temperature, animal and human presence, and a brief description of each site.

Additional file 5: Supplementary Table S2. Primers for molecular identification of sand flies.

Additional file 6: Supplementary Table S3. GenBank ID of individual sand flies analyzed.

Additional file 7: Supplementary Table S4. *COI* haplotypes of sand flies were used to construct the TCS network.

## Data Availability

The nucleotide sequences obtained in this study were deposited in the GenBank™ database (https://www.ncbi.nlm.nih.gov/nuccore) as provided in supplementary file Table S3.

## Abbreviations

BLASTN: Basic local alignment search tool for nucleotide
BOLD: Barcode of Life Database
CDC: Center for Diseases Control and Prevention
*COI*: Cytochrome oxidase *c* subunit I
*Cytb*: Cytochrome *b*
PCR: Polymerase chain reaction
SR: Species richness
