# Supplementary figures and images for: Survey of sand fly fauna in six provinces of Southern Vietnam with species identification using DNA barcoding
Source: Parasit Vectors. 2024 Oct 29;17:443. doi: 10.1186/s13071-024-06509-w (PMC11523761; doi:10.1186/s13071-024-06509-w)

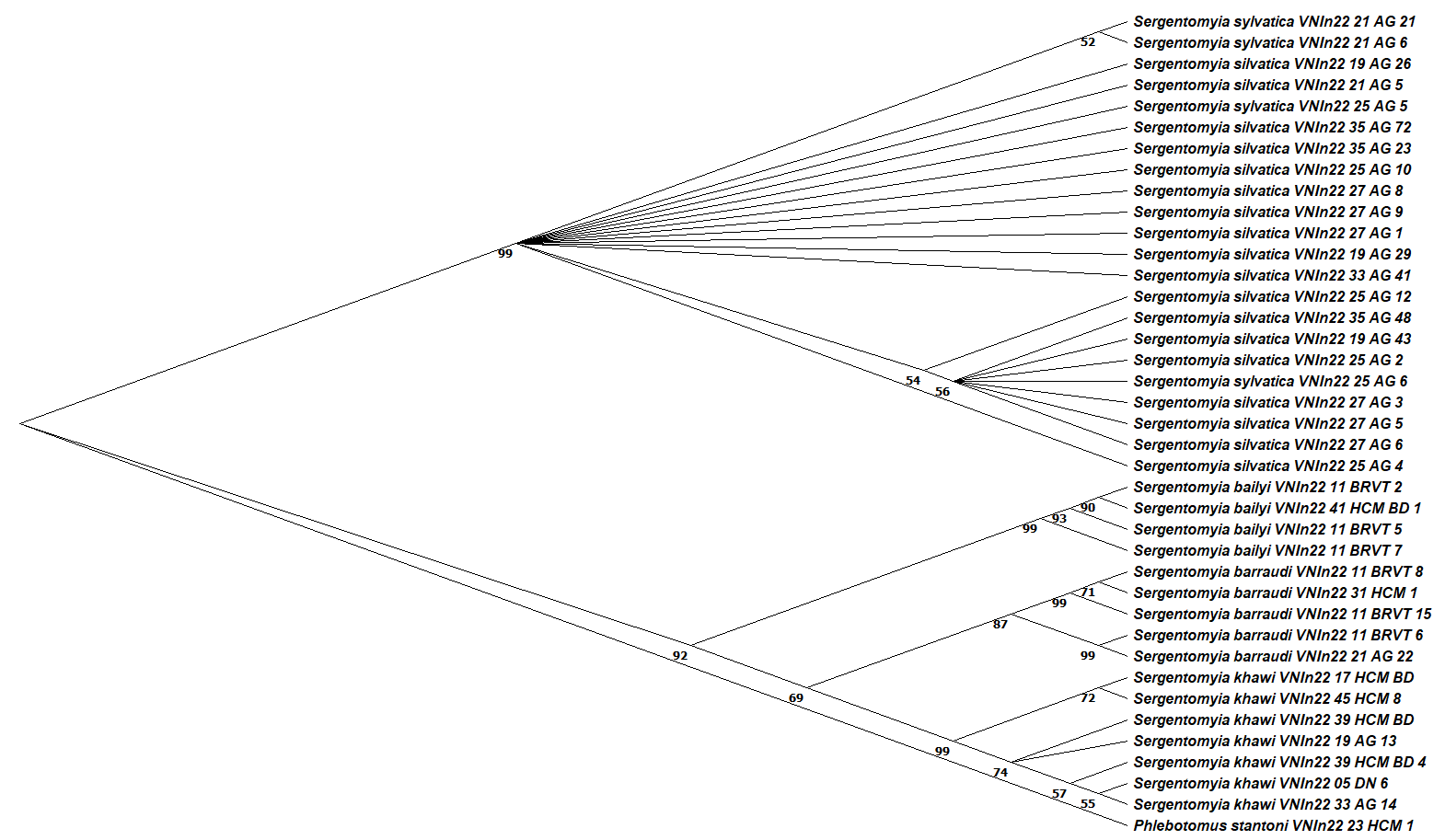

Supplement: Supplementary file 2 — Additional file 2: Supplementary Figure S2. The evolutionary relationships of taxa based on COI sequences were inferred using the neighbor-joining method. The percentage of replicate trees in which the associated taxa clustered together during the bootstrap test (1000 replicates) is shown above the branches. The evolutionary distances were calculated using the Kimura 2-Parameter method [13] and are expressed as the number of base substitutions per site. The following letter codes represent the sampling sites: AG: An Giang, DN: Dong Nai, HCM: Ho Chi Minh, TN: Tay Ninh, BD: Binh Duong, BRVT: Ba Ria-Vung Tau. [file 13071_2024_6509_MOESM2_ESM.tif]

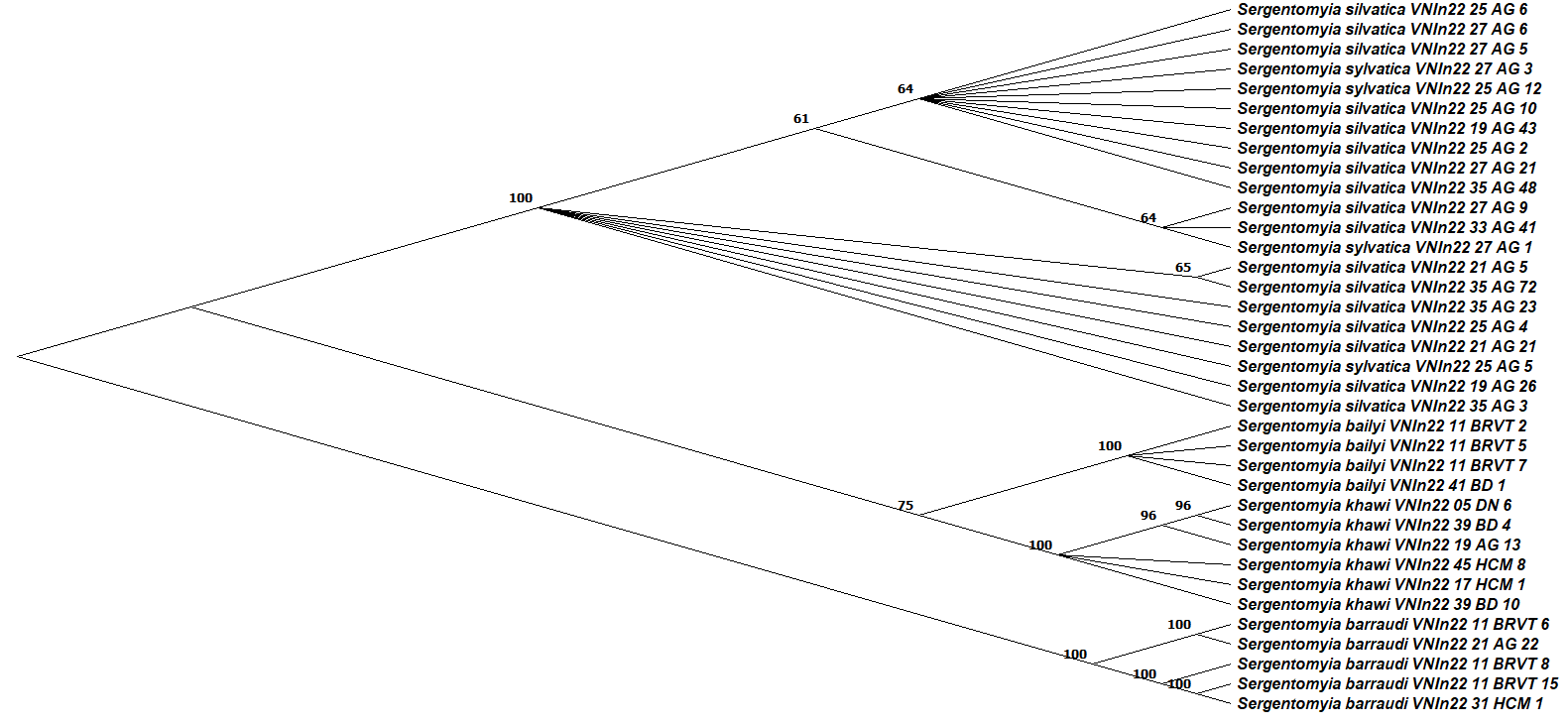

Supplement: Supplementary file 3 — Additional file 3: Supplementary Figure S3. The evolutionary relationships of taxa based on cytb sequences were inferred using the neighbor-joining method. The percentage of replicate trees in which the associated taxa clustered together during the bootstrap test (1000 replicates) is shown above the branches. The evolutionary distances were calculated using the Kimura 2-Parameter method [13] and are expressed as the number of base substitutions per site. The following letter codes represent the sampling sites: AG: An Giang, DN: Dong Nai, HCM: Ho Chi Minh, TN: Tay Ninh, BD: Binh Duong, BRVT: Ba Ria-Vung Tau. [file 13071_2024_6509_MOESM3_ESM.tif]
