# Supplementary material for: Survey of sand fly fauna in six provinces of Southern Vietnam with species identification using DNA barcoding
Source: Parasit Vectors. 2024 Oct 29;17:443. doi: 10.1186/s13071-024-06509-w (PMC11523761; doi:10.1186/s13071-024-06509-w)
Supplement: Supplementary file 5 — Additional file 5: Supplementary Table S2. Primers for molecular identification of sand flies. [file 13071_2024_6509_MOESM5_ESM.docx]

**Supplementary Table S2** Primers for molecular identification of sand flies identification

| Target gene | Primer Name | Primer sequence (5’-3') | Target size (bp) | Amplification Protocol | Reference |
| --- | --- | --- | --- | --- | --- |
| *Cytb* | Sandfly_Cytb_F_N1N_PDR  Sandfly_Cytb_R_C3B_PDR | CAYATTCAACCWGAATGATA  GGTAYWTTGCCTCGAWTTCGWTATGA | 500 | 94^o^C: 3 min;  5 cycles: 94^o^C: 1 min,  40^o^C: 1 min; 68^o^C: 1 min  35 cycles: 94^o^C: 1 min,  44^o^C: 1 min; 68^o^C: 1 min;  68 ^o^C: 10 min | Esseghir et al. (1997) |
|  |  |  |  |  |  |
| *Cox1* | SandflyCOX1F_LepF  SandflyCOX1R_LepR | ATTCAACCAATCATAAAGATATTGG  AAACTTCTGATGTCCAAAAAATCA | 708 | 94^o^C: 3 min;  5 cycles: 94^o^C: 1 min,  45^o^C: 90 sec; 86^o^C: 1 min  35 cycles: 94^o^C: 1 min,  51^o^C: 90 sec; 86^o^C: 1 min;  68 ^o^C: 10 min | Depaquit et al. (2009) |
|  |  |  |  |  |  |
