# Supplementary material for: Survey of sand fly fauna in six provinces of Southern Vietnam with species identification using DNA barcoding
Source: Parasit Vectors. 2024 Oct 29;17:443. doi: 10.1186/s13071-024-06509-w (PMC11523761; doi:10.1186/s13071-024-06509-w)
Supplement: Supplementary file 6 — Additional file 6: Supplementary Table S3. GenBank ID of individual sand flies analyzed. [file 13071_2024_6509_MOESM6_ESM.docx]

**Supplementary Table S3** GenBank ID of individual sand flies analyzed.

| **No.** | **Species** | **Sequence ID** | **GenBank Accession number** | | | **Collection date** | | **Lat -Lon** |
| --- | --- | --- | --- | --- | --- | --- | --- | --- |
|  |  |  | ***cytb*** | ***COI*** |  | |  | |
| 1 | *Phlebotomus stantoni* | VNIn22_23_HCM_1 | N/A | OR044839 | 29 Jun 2022 | | 10.8255, 106.6792 | |
| 2 | *Sergentomyia khawi* | VNIn22_05_DN_6 | OR051794 | OR044840 | 17 May 2022 | | 11.4223, 107.4291 | |
| 3 | *Sergentomyia khawi* | VNIn22_17_HCM_BD_1 | OR051796 | OR044841 | 30 May 2022 | | 10.8606, 106.7723 | |
| 4 | *Sergentomyia khawi* | VNIn22_19_AG_13 | OR051793 | OR044842 | 5 Jul 2022 | | 10.4671, 109.9364 | |
| 5 | *Sergentomyia khawi* | VNIn22_45_HCM_8 | OR051798 | OR044843 | 21 May 2022 | | 11.0335, 106.4135 | |
| 6 | *Sergentomyia khawi* | VNIn22_39_HCM_BD_10 | OR051797 | OR044844 | 31 May 2022 | | 10.9085, 106.7701 | |
| 7 | *Sergentomyia khawi* | VNIn22_39_HCM_BD_4 | OR051795 | OR044845 | 31 May 2022 | | 10.9085, 106.7701 | |
| 8 | *Sergentomyia khawi* | VNIn22_33_AG_14 | N/A | OR044878 | 9 Jun 2022 | | 10.4507, 104.9024 | |
| 9 | *Sergentomyia khawi* | VNIn22_01_TN_12 | N/A | PP708919 | 14 May 2022 | | 11.0335, 106.4135 | |
| 10 | *Sergentomyia khawi* | VNIn22_03 _TN_3 | N/A | PP708920 | 14 May 2022 | | 11.0335, 106.4135 | |
| 11 | *Sergentomyia khawi* | VNIn22_03_TN_8 | N/A | PP708921 | 14 May 2022 | | 11.0335, 106.4135 | |
| 12 | *Sergentomyia khawi* | VNIn22_05_DN_5 | N/A | PP708922 | 17 May 2022 | | 11.4223, 107.4291 | |
| 13 | *Sergentomyia khawi* | VNIn22_11_BRVT_1 | N/A | PP708923 | 21 May 2022 | | 10.3606, 107.0612 | |
| 14 | *Sergentomyia khawi* | VNIn22_03_TN_1 | N/A | PP708616 | 14 May 2022 | | 11.0335, 106.4135 | |
| 15 | *Sergentomyia bailyi* | VNIn22_11_BRVT_2 | OR051800 | OR044846 | 21 May 2022 | | 10.3606, 107.0612 | |
| 16 | *Sergentomyia bailyi* | VNIn22_11_BRVT_5 | OR051801 | OR044847 | 21 May 2022 | | 10.3606, 107.0612 | |
| 17 | *Sergentomyia bailyi* | VNIn22_11_BRVT_7 | OR051802 | OR044848 | 21 May 2022 | | 10.3606, 107.0612 | |
| 18 | *Sergentomyia bailyi* | VNIn22_41_HCM_BD_1 | OR051799 | OR044849 | 29 May 2022 | | 10.8927, 106.7831 | |
| 19 | *Sergentomyia barraudi* | VNIn22_11_BRVT_8 | OR051805 | OR044850 | 21 May 2022 | | 10.3606, 107.0612 | |
| 20 | *Sergentomyia barraudi* | VNIn22_11_BRVT_6 | OR051803 | OR044851 | 21 May 2022 | | 10.3606, 107.0612 | |
| 21 | *Sergentomyia barraudi* | VNIn22_11_BRVT_15 | OR051806 | OR044852 | 21 May 2022 | | 10.3606, 107.0612 | |
| 22 | *Sergentomyia barraudi* | VNIn22_31_HCM_1 | OR051807 | OR044853 | 29 Jun 2022 | | 10.8255, 106.6792 | |
| 23 | *Sergentomyia barraudi* | VNIn22_21_AG_22 | OR051804 | OR044854 | 5 Jul 2022 | | 10.4671, 109.9364 | |
| 24 | *Sergentomyia barraudi* | VNIn22_05_DN_8 | N/A | PP708924 | 17 May 2022 | | 11.4223, 107.4291 | |
| 25 | *Sergentomyia barraudi* | VNIn22_49_HCM_6 | PP719692 | PP708925 | 30 May 2022 | | 11.03543, 106.42898 | |
| 26 | *Sergentomyia silvatica* | VNIn22_19_AG_26 | OR051812 | OR044855 | 5 Jul 2022 | | 10.4671, 109.9364 | |
| 27 | *Sergentomyia silvatica* | VNIn22_19_AG_29 | N/A | OR044856 | 5 Jul 2022 | | 10.4671, 109.9364 | |
| 28 | *Sergentomyia silvatica* | VNIn22_19_AG_43 | OR051813 | OR044857 | 5 Jul 2022 | | 10.4671, 109.9364 | |
| 29 | *Sergentomyia silvatica* | VNIn22_21_AG_21 | OR051808 | OR044858 | 5 Jul 2022 | | 10.4671, 109.9364 | |
| 30 | *Sergentomyia silvatica* | VNIn22_21_AG_5 | OR051823 | OR044859 | 5 Jul 2022 | | 10.4671, 109.9364 | |
| 31 | *Sergentomyia silvatica* | VNIn22_21_AG_6 | N/A | OR044860 | 5 Jul 2022 | | 10.4671, 109.9364 | |
| 32 | *Sergentomyia silvatica* | VNIn22_25_AG_2 | OR051811 | OR044861 | 10 Jun 2022 | | 10.4507, 104.9024 | |
| 33 | *Sergentomyia silvatica* | VNIn22_25_AG_4 | OR051827 | OR044862 | 10 Jun 2022 | | 10.4507, 104.9024 | |
| 34 | *Sergentomyia silvatica* | VNIn22_25_AG_6 | OR051814 | OR044863 | 10 Jun 2022 | | 10.4507, 104.9024 | |
| 35 | *Sergentomyia silvatica* | VNIn22_25_AG_5 | OR051828 | OR044864 | 10 Jun 2022 | | 10.4507, 104.9024 | |
| 36 | *Sergentomyia silvatica* | VNIn22_25_AG_10 | OR051815 | OR044865 | 10 Jun 2022 | | 10.4507, 104.9024 | |
| 37 | *Sergentomyia silvatica* | VNIn22_25_AG_12 | OR051816 | OR044866 | 10 Jun 2022 | | 10.4507, 104.9024 | |
| 38 | *Sergentomyia silvatica* | VNIn22_27_AG_1 | OR051817 | OR044867 | 10 Jun 2022 | | 10.4507, 104.9024 | |
| 39 | *Sergentomyia silvatica* | VNIn22_27_AG_3 | OR051818 | OR044868 | 10 Jun 2022 | | 10.4507, 104.9024 | |
| 40 | *Sergentomyia silvatica* | VNIn22_27_AG_5 | OR051819 | OR044869 | 10 Jun 2022 | | 10.4507, 104.9024 | |
| 41 | *Sergentomyia silvatica* | VNIn22_27_AG_6 | OR051820 | OR044870 | 10 Jun 2022 | | 10.4507, 104.9024 | |
| 42 | *Sergentomyia silvatica* | VNIn22_33_AG_41 | OR051809 | OR044871 | 9 Jun 2022 | | 10.4507, 104.9024 | |
| 43 | *Sergentomyia silvatica* | VNIn22_35_AG_72 | OR051825 | OR044872 | 9 Jun 2022 | | 10.4507, 104.9024 | |
| 44 | *Sergentomyia silvatica* | VNIn22_35_AG_48 | OR051810 | OR044873 | 9 Jun 2022 | | 10.4507, 104.9024 | |
| 45 | *Sergentomyia silvatica* | VNIn22_35_AG_3 | OR051824 | OR044875 | 9 Jun 2022 | | 10.4507, 104.9024 | |
| 46 | *Sergentomyia silvatica* | VNIn22_27_AG_8 | N/A | OR044876 | 10 Jun 2022 | | 10.4507, 104.9024 | |
| 47 | *Sergentomyia silvatica* | VNIn22_27_AG_9 | OR051822 | OR044877 | 10 Jun 2022 | | 10.4507, 104.9024 | |
| 48 | *Sergentomyia silvatica* | VNIn22_05_DN_12 | PP719693 | PP708926 | 17 May 2022 | | 11.4223, 107.4291 | |

**Note:** N/A indicates not available
