# Supplementary material for: Survey of sand fly fauna in six provinces of Southern Vietnam with species identification using DNA barcoding
Source: Parasit Vectors. 2024 Oct 29;17:443. doi: 10.1186/s13071-024-06509-w (PMC11523761; doi:10.1186/s13071-024-06509-w)
Supplement: Supplementary file 7 — Additional file 7: Supplementary Table S4. COI haplotypes of sand flies were used to construct the TCS network. [file 13071_2024_6509_MOESM7_ESM.docx]

**Supplementary Table S4** Sand fly’s COI Haplotypes used to construct the TCS network

| **Haplotype** | **Sequences ID/ Accession number** | **Sampling location** | **Species** |
| --- | --- | --- | --- |
| H1 | OK576209 | Thailand | *Se. barraudi* |
| H2 | MZ400954 | Thailand | *Se. barraudi* |
| H3 | MZ400940 | Thailand | *Se. barraudi* |
| H4 | MZ400912 | Thailand | *Se. barraudi* |
| H5 | MN850828 | Thailand | *Se. barraudi* |
| H6 | MN850827 | Thailand | *Se. barraudi* |
| H7 | LC136902 | Thailand | *Se. barraudi* |
| H8 | OK576211 | Thailand | *Se. khawi* |
| H9 | OP879808  OP879807 | Thailand | *Se. silvatica* |
| H10 | JSTH046-16 | Thailand | *Se. silvatica* |
| H11 | OR044850  PP708924  PP708925 | Ba Ria Vung Tau  Dong Nai  Ho Chi Minh | *Se. barraudi* |
| H12 | OR044851 | Ba Ria Vung Tau | *Se. barraudi* |
| H13 | OR044852 | Ba Ria Vung Tau | *Se. barraudi* |
| H14 | OR044853 | Ho Chi Minh | *Se. barraudi* |
| H15 | OR044854 | An Giang | *Se. barraudi* |
| H16 | OR044840  OR044878 | Dong Nai  An Giang | *Se. khawi* |
| H17 | OR044841 | Binh Duong | *Se. khawi* |
| H18 | OR044842 | An Giang | *Se. khawi* |
| H19 | PP708616  OR044843 | Tay Ninh  Ho Chi Minh | *Se. khawi* |
| H20 | OR044844 | Tay Ninh | *Se. khawi* |
| H21 | PP708921  OR044845 | Tay Ninh  Binh Duong | *Se. khawi* |
| H22 | PP708919 | Tay Ninh | *Se. khawi* |
| H23 | PP708920 | Tay Ninh | *Se. khawi* |
| H24 | PP708922 | Dong Nai | *Se. khawi* |
| H25 | PP708923 | Ba Ria Vung Tau | *Se. khawi* |
| H26 | OR044855 | An Giang | *Se. silvatica* |
| H27 | OR044856, OR044865  OR044871, OR044871 | An Giang | *Se. silvatica* |
| H28 | OR044857, OR044861  OR044863, OR044866  OR044868, OR044869  OR044870 | An Giang | *Se. silvatica* |
| H29 | OR044858 | An Giang | *Se. silvatica* |
| H30 | OR044859, OR044864  OR044872, OR044874 | An Giang | *Se. silvatica* |
| H31 | OR044860 | An Giang | *Se. silvatica* |
| H32 | OR044862 | An Giang | *Se. silvatica* |
| H33 | OR044867 | An Giang | *Se. silvatica* |
| H34 | OR044873 | An Giang | *Se. silvatica* |
| H35 | OR044877 | An Giang | *Se. silvatica* |
| H36 | PP708926 | Dong Nai | *Se. silvatica* |
| H37 | KT284883 | Sri Lanka | *Se. bailyi* |
| H38 | MF966720 | China | *Se. bailyi* |
| H39 | HQ585383 | India | *Se. bailyi* |
| H40 | LC136900 | Thailand | *Se. bailyi* |
| H41 | MF966711 | Thailand | *Se. bailyi* |
| H42 | OR044846 | Ba Ria Vung Tau | *Se. bailyi* |
| H43 | OR044847 | Ba Ria Vung Tau | *Se. bailyi* |
| H44 | OR044848 | Ba Ria Vung Tau | *Se. bailyi* |
| H45 | OR044849 | Binh Duong | *Se. bailyi* |
| H46 | LC136901 | Thailand | *Se. bailyi* |
| H47 | KT284881 | Sri Lanka | *Se. bailyi* |
| H48 | HQ585386 | India | *Se. bailyi* |
| H49 | OP879774 | Thailand | *Se. khawi* |
